# Comparative analysis of the immune responses of *Cc*IgZ3 in mucosal tissues and the co-expression of *Cc*IgZ3 and PCNA in the gills of common carp (*Cyprinus carpio L.*) in response to TNP-LPS

**DOI:** 10.1186/s12917-023-03854-3

**Published:** 2024-01-06

**Authors:** Jiaqi Zhang, Haoyue Ren, Qiannan Zhu, Xiangrui Kong, Feng Zhang, Chang Wang, Yimeng Wang, Guiwen Yang, Fumiao Zhang

**Affiliations:** 1 Key Laboratory of Animal Resistance Biology of Shandong Province, College of Life Sciences, Shandong Normal University, 88 East Wenhua Road, Jinan, Shandong 250014 China; 2 School of Basic Medical Sciences, Shandong First Medical University & Shandong Academy of Medical Science, Jinan, Shandong 250117 China

**Keywords:** Common carp, *Cc*IgZ3, Mucosal tissues, Immune responses, Proliferation

## Abstract

Fish live in an aquatic environment rich in various microorganisms and pathogens. Fish mucosal-associated lymphoid tissue (MALT) plays a very important role in immune defence. This study was conducted to characterize the immune response mediated by *Cc*IgZ3 in common carp (*Cyprinus carpio.*) and investigate the proliferating *Cc*IgZ3^+^ B lymphocytes in gill. We determined the expression of *Cc*IgZ3 in many different tissues of common carp following stimulation by intraperitoneal injection of TNP-LPS (2,4,6-Trinitrophenyl hapten conjugated to lipopolysaccharide) or TNP-KLH (2,4,6-Trinitrophenyl hapten conjugated to Keyhole Limpet Hemocyanin). Compared with TNP-KLH, TNP-LPS can induce greater *Cc*IgZ3 expression in the head kidney, gill and hindgut, especially in the gill. The results indicate that the gill is one of the main sites involved in the immune response mediated by *Cc*IgZ3. To examine the distribution of *CcIgZ3*^+^ B lymphocytes, immunohistochemistry (IHC) experiments were performed using a polyclonal antibody against *Cc*IgZ3. The results indicated that *Cc*IgZ3 was detected in the head kidney, hindgut and gill. To further examine whether *CcIgZ3*^+^ B lymphocytes proliferate in the gills, proliferating *CcIgZ3*^+^ B cells were analysed by immunofluorescence staining using an anti-*Cc*IgZ3 polyclonal antibody and an anti-PCNA monoclonal antibody. *Cc*IgZ3 and PCNA (Proliferating Cell Nuclear Antigen) double-labelled cells in the gills were located within the epithelial cells of the gill filaments of common carp stimulated with TNP-LPS at 3 dps and 7 dps, and relatively more proliferating *CcIgZ3*^+^ B cells appeared in the gills of common carp at 7 dps. These data imply that *Cc*IgZ3^+^ B cells in the gills might be produced by local proliferation following TNP-LPS stimulation. In summary, compared with those in TNP-KLH, *Cc*IgZ3 preferentially affects the gills of common carp following challenge with TNP-LPS. *Cc*IgZ3^+^ B cells proliferate in the gills to quickly produce the *Cc*IgZ3 antibody. In addition, *Cc*IgZ3^+^ B cells can be activated to induce a strong immune response very early locally in the gill and produce the antibody *Cc*IgZ3, which helps exert an immune-protective effect. These results suggest that an effective vaccine can be designed to promote production of the mucosal antibody *Cc*IgZ3.

## Introduction

Fish live in aquatic environments for a long time, where water is rich in microorganisms, including various bacteria and viruses. When fish are exposed to this environment for a long time, the situation they face is more complex than that of animals living on land. To protect against various pathogens, fish have evolved innate immunity and adaptive immunity. Since most of these pathogens invade the body of fish through mucosal epithelial barriers, such as the skin, gill and intestine [1, 2], an adaptive immune system involving the mucosal body surface of fish plays an important role. Mucosal-associated lymphoid tissue (MALT) in fish mainly includes gut-associated lymphoid tissue (GALT), skin-associated lymphoid tissue (SALT), gill-associated lymphoid tissue (GIALT), nasal-associated lymphoid tissue (NALT), buccal-associated lymphoid tissue and pharyngeal-associated lymphoid tissue [3]. Unlike mammals, which also have organized MALTs (O-MALTs), such as Peyer’s patches and the amygdala, fish contain only diffuse MALTs (D-MALTs) [2]. Many studies have shown that these MALT lymphomas produce physiologically specific immune responses to defend against pathogen invasion after infection [4–7]. Fish were the first organisms to develop immunoglobulins in the body, and there are three main types of Ig in bony fish: IgM, IgD, and IgT/IgZ [8, 9]. IgM mainly affects the serum but has a decreased titre in mucosal tissues [10]. Studies have shown that the total IgM concentration and parasite-specific IgM binding are significantly increased in the serum of trout infected with parasites, and the proliferation of IgM^+^ B cells is increased in the head kidney [11, 12]. Additionally, different concentrations of IgM have been shown to appear as tetramers in different types of mucus in rainbow trout (*Oncorhynchus mykiss*) [3]. IgD has been found to be expressed with IgM in rainbow trout B cells [13], but whether IgD plays a role in mucosal tissue immunity is unknown. IgT/IgZ, which is composed of four constant regions, was reported by Danilova et al. in 2005 [8] and Hu et al. in 2009 [14]; these authors first discovered secreted and membrane-bound IgZ in zebrafish (*Danio rerio*). Since then, an increasing number of IgZ/IgT subtypes have been reported, including IgZ1, IgZ2 and *CcIgZ3* in common carp and IgT1, IgT2, IgT3 and IgT4 in stickback fishes (*Notacanthidae*) [15]. All bony fish except catfish (*Silurus asotus*) and killifish (*Oryzias latipes*) express IgT/IgZ, and IgT/IgZ are the main forces that drive mucosal immunity [16].

IgT and IgZ have been reported to be involved in intestinal [4, 5] and gill [9, 17, 18] mucosal immunity in many bony fishes. Studies have shown that IgT is the main gill immunoglobulin that is involved in the pathogen-specific immune response in rainbow trout, and the gill microflora is generally wrapped by IgT [13]. Furthermore, the expression of three subclasses of IgT1, IgT2 and IgT3 in rainbow trout was analysed after stimulation with LPS from *E. coli* [4]. In studies of rainbow trout immunity to parasitic trophotrophotes, the expression of genes encoding IgT and IgM was significantly increased [7]. The abundance of IgT^+^ cells in the gill epithelium after parasitic infection suggested that these cells play an important role in the defence against pathogen invasion [13]. According to a study by Zhen Xu et al., sIgT is often polymerized in gill mucus, and the content of sIgT in gill mucus is approximately 5 times greater and 4 times lower than that reported in the skin and intestinal mucus, respectively. The IgD/IgM and IgT/IgM ratios in gill mucus were 10 and 100 times greater than those in trout serum, respectively. The ratio of IgT/IgM in branchial mucus was significantly increased, suggesting the potential role of IgT in branchial immunity. However, the mechanism and role of IgT/IgZ in the gills of common carp remain unclear.

As the breathing organ of fish, gills are easily invaded by pathogens. Therefore, gill-associated lymphoid tissue is a very important site in the mucosal immunity of bony fishes. Gill tissue contains many innate and adaptive immune cells, and several innate and adaptive immune molecules and pathways are activated in gills [19–22].

Common carp are important aquaculture fish in our country, and disease control in common carp aquaculture is highly important. Currently, there are three known subtypes of IgZ antibodies in carp, but the immune response in different tissues is still unclear. In addition, studies on IgZ-secreting B-cell proliferation and differentiation are rare, which limits further study of the maturation and function of B cells in common carp. In this study, the expression of *Cc*IgZ3 in mucosal tissues and the colocalization of *Cc*IgZ3 and PCNA in the gills of common carp stimulated with TNP-LPS were analysed. We hope that this study will be helpful for providing an effective method of vaccine design that can induce production of the mucosal antibody *Cc*IgZ3.

## Materials and methods

### Ethics statement

For all the experiments on live animals, we confirmed that the methods were carried out in accordance with the relevant guidelines and regulations. The protocol was approved by the Animal Experimental Ethics Committee of Shandong Normal University (Permit Number: AEECSDNU2021017). All efforts were made to minimize suffering. The study was carried out in compliance with the ARRIVE guidelines.

### Experimental animals

Common carp (*Cyprinus. carpio*) with a mean weight of 150 g were obtained from a local fish farm and maintained in aquarium tanks with aerated recirculating tap water at 22–25 °C. The fish were acclimated to the aquarium tanks for at least 2 weeks before being used in the experiments and were fed daily with commercial pellets. The feeding was terminated 48 h prior to sacrifice.

### Stimulation with TNP-LPS and TNP-KLH and sample preparation

Nine groups were established, and the T-cell-independent (TI) antigen group was divided into four groups. The fish were intraperitoneally injected with TNP-LPS at a concentration of 40 µg/ml and sampled at 1, 3, 7 and 15 days post stimulation (dps). The T-cell-dependent (TD) antigen groups were divided into four groups; the fish in these groups were intraperitoneally injected with TNP-KLH and sampled at 1, 3, 7 and 15 dps. Fish in the control group were intraperitoneally injected with TNP-PBS. The head kidney, gill, liver, hindgut and skin were sampled at 1 day, 3 days, 7 days and 15 days after injection.

### Total RNA extraction and quantitative real-time PCR (q-PCR) analysis

Fish were anaesthetized by immersion in a solution of tricaine (MS222, Sigma Aldrich) and sacrificed. Tissue samples of the head kidney, gill, liver, hindgut, and skin were collected immediately, frozen in liquid nitrogen, and stored at -80 °C until use. Each frozen sample was ground in a mortar with nitrogen, and total RNA was subsequently isolated using TRIzol Universal reagent (Tiangen, China). The quantity and quality of the total RNA were assessed using a NanoDrop Spectrophotometer (Thermo Scientific, USA) for all the samples. First-strand cDNA was synthesized from 2 µg of total RNA using the FastQuant RT Kit (with gDNase) (Tiangen, China) according to the manufacturer’s instructions. Total RNA was extracted from the collected samples following the procedure, and cDNA was subsequently stored at -80 °C until use in quantitative real-time PCR (qRT‒PCR). PCR was conducted with the specific primers *CcIgZ3*-qF3 (TGCGGACTCGCTGACTTTTA) and *CcIgZ3*-qR3 (GACAGGAACCAAGCTCAGGG) with SuperReal PreMix Plus (SYBR Green, Tiangen, China). The qRT‒PCR amplification program was as follows: 94 °C for 30 s, 55 °C for 30 s, and 72 °C for 30 s for 30 cycles. The qRT‒PCR amplification programme for ribosomal protein *S11* (as a standard) was 94 °C for 30 s, 55 °C for 30 s, and 72 °C for 30 s for 30 cycles using the primers *S11*qF (CCGTGGGTGACATCGTTACA) and *S11*qR (TCAGGACATTGAACCTCACTGTCT) in parallel tubes. The relative expression of the immunoglobulin gene was calculated and normalized to ribosomal protein *S11*.

### Immunohistochemical detection of *Cc*IgZ3 in the head kidney, gill and hindgut of common carp using a polyclonal antibody against CcIgZ3

After the carp were dissected, the head kidney, gill and hindgut tissues were collected. The tissues were cleaned with PBS, soaked in 4% paraformaldehyde, fixed at room temperature for 24 h, dehydrated, cleared and embedded in paraffin. The tissue sections were 4 μm long and stained with H&E. Prior staining was used to evaluate the quality of the sections before immunohistochemical staining. Briefly, the immunohistochemistry procedure was as follows: the slices were transferred to xylene for paraffin penetration 3 times for 10 min and then rehydrated in a series of ethanol solutions supplemented with 100% ethanol for 10 min×2, transferred to 95% ethanol and 75% ethanol for 5 min, and finally soaked in deionized water for 5 min. The sections were dewaxed in 10 mM EDTA (pH 8.0) for 15 min, cooled, and incubated with 3% H_2_O_2_ for 10 min. After being washed with pure water twice and with PBST (0.1% Tween-20) for 5 min, the sections were blocked with 5% rabbit serum (Solarbio, China) for 1 h and incubated overnight with a rabbit anti-*Cc*IgZ3 polyclonal antibody (1:200, Abmart, China) and control sera. The sections were washed in PBST three times and then incubated with 1:200 diluted, biotin-conjugated goat anti-rabbit Ab for 1 h. Finally, HRP-streptavidin was added after washing twice, and the samples were subsequently washed and developed with the DAB substrate 3,3-n-diaminobenzidine tetrahydrochloride. Slides were lightly counterstained with haematoxylin [23].

### Immunofluorescence colocalization of *Cc*IgZ3 and PCNA in the gills of common carp after stimulation with TNP-LPS

The procedures for tissue sampling, fixation, embedding, sectioning, dewaxing and rehydration were the same as those described above. The slices were put into 1× sodium citrate repair solution, boiled at 95 °C for 15 min, and then cooled to room temperature. The tissues were washed with PBST (PBS + 2% Triton X-100) for 30 min. Circles were drawn around the tissues, which were blocked using the appropriate serum (PBST diluted 20 times) and incubated at room temperature for 1 h. The primary rabbit anti-*Cc*IgZ3 polyclonal antibody (1:100 dilution) and mouse anti-PCNA monoclonal antibody (1:200 dilution; ET111201; Huabio, China) were added, rabbit IgG and mouse IgG (1:500 dilution; Beyotime, China) were added as isotype controls, and blocking solution was added as the negative control. After incubating at 4 °C overnight, the slices were kept at room temperature for 30 min and then washed with PBST. Alexa Fluor 555-labelled donkey anti-mouse (1:1000, Abcam) antibody and Alexa Fluor 488-labelled goat anti-rabbit (1:1000, Abcam) antibody were used as secondary antibodies for labelling and samples were incubated for 1 h in the dark at 4 °C with gentle shaking. After being washed with PBST, the slices were stained with DAPI. Mounting was performed with coverslips (CITOTEST) and Antifade Mounting Medium. The slides were examined under an LSM confocal microscope (Zeiss, Germany), and images were taken.

## Results

### Quantitative RT‒PCR

**Fig. 1:**
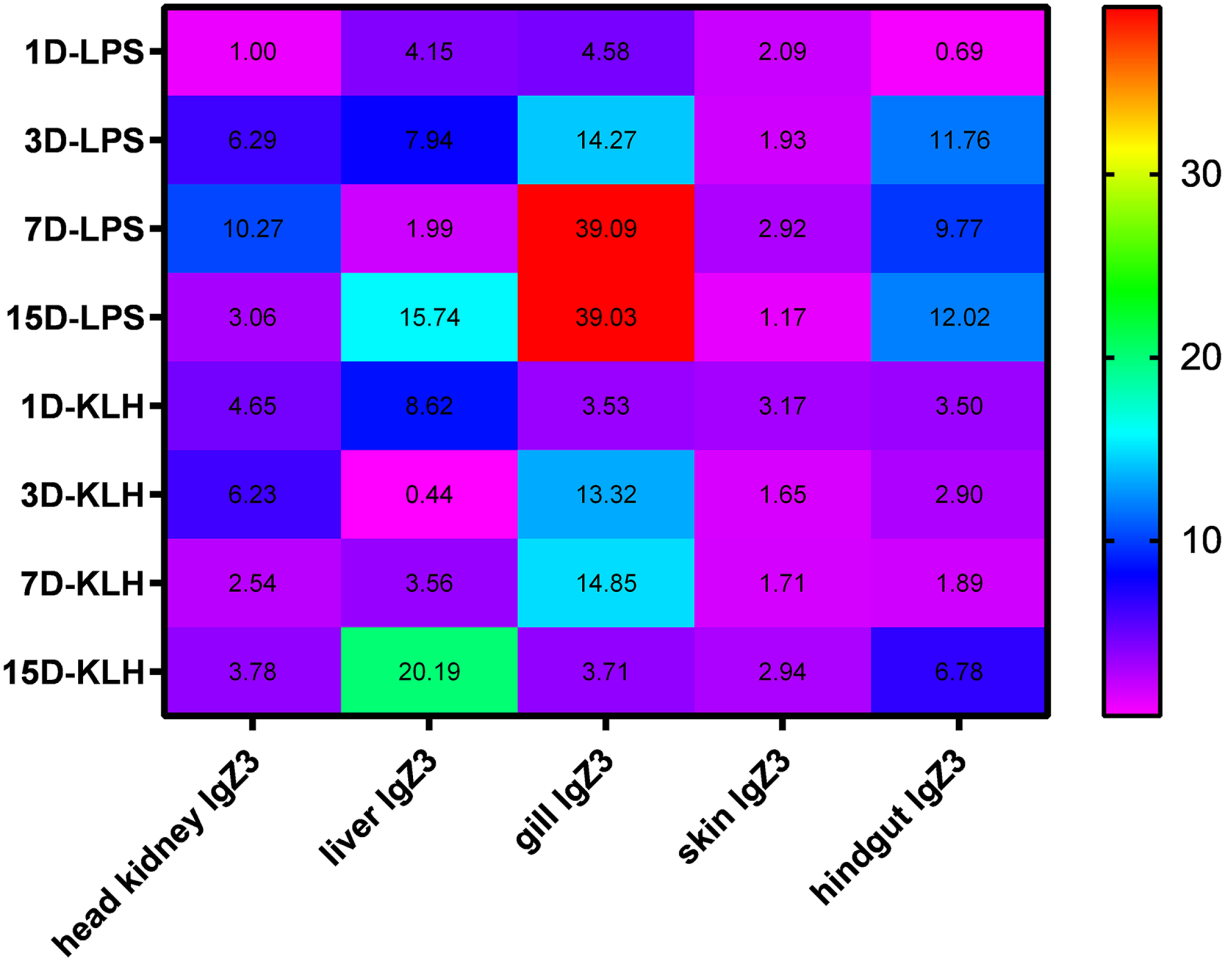
Detection of *CcIgZ3* expression in different tissues from common carp stimulated with TNP-LPS or TNP-KLH at 1, 3, 7 and 15 days post stimulation (dps)

Both TI and TD can activate the *CcIgZ3* response in different tissues to varying degrees. Compared with TNP-KLH, TNP-LPS can induce greater *CcIgZ3* expression in the head kidney, gill and hindgut, especially in the gill. The fold changes in *CcIgZ3* expression in the gills on days 1, 3, 7 and 15 after TNP-LPS stimulation were greater than those after TNP-KLH stimulation. The fold change in *CcIgZ3* expression in the gills reached 4.58, 14.27, 39.09 and 39.03 on days 1, 3, 7 and 15 after TNP-LPS stimulation, respectively. In the TNP-KLH stimulation group, the fold change in *CcIgZ3* expression in the gill was also greater than that in the head kidney, liver, skin and hindgut at 1, 3 and 7 dps (except at 15 days). These results indicate that the gill is one of the main sites involved in the *CcIgZ3* immune response. Both TI and TD immune responses can effectively induce an increase in *CcIgZ3* expression in the gill, and *CcIgZ3* is involved in the gill mucosal immune response in carp. In addition, the results showed that in the liver, the TNP-KLH-induced increase in *CcIgZ3* expression was greater than that in the control group, and the fold change reached 20.19 15 days after stimulation. Neither the TI nor the TD antigen induced a greater *CcIgZ3* response in the skin, with fold changes ranging from 1.17 to 3.17 (Fig. 1).

### Immunohistochemical detection of *Cc*IgZ3^+^ B lymphocytes in different immune-related tissues of common carp

**Fig. 2:**
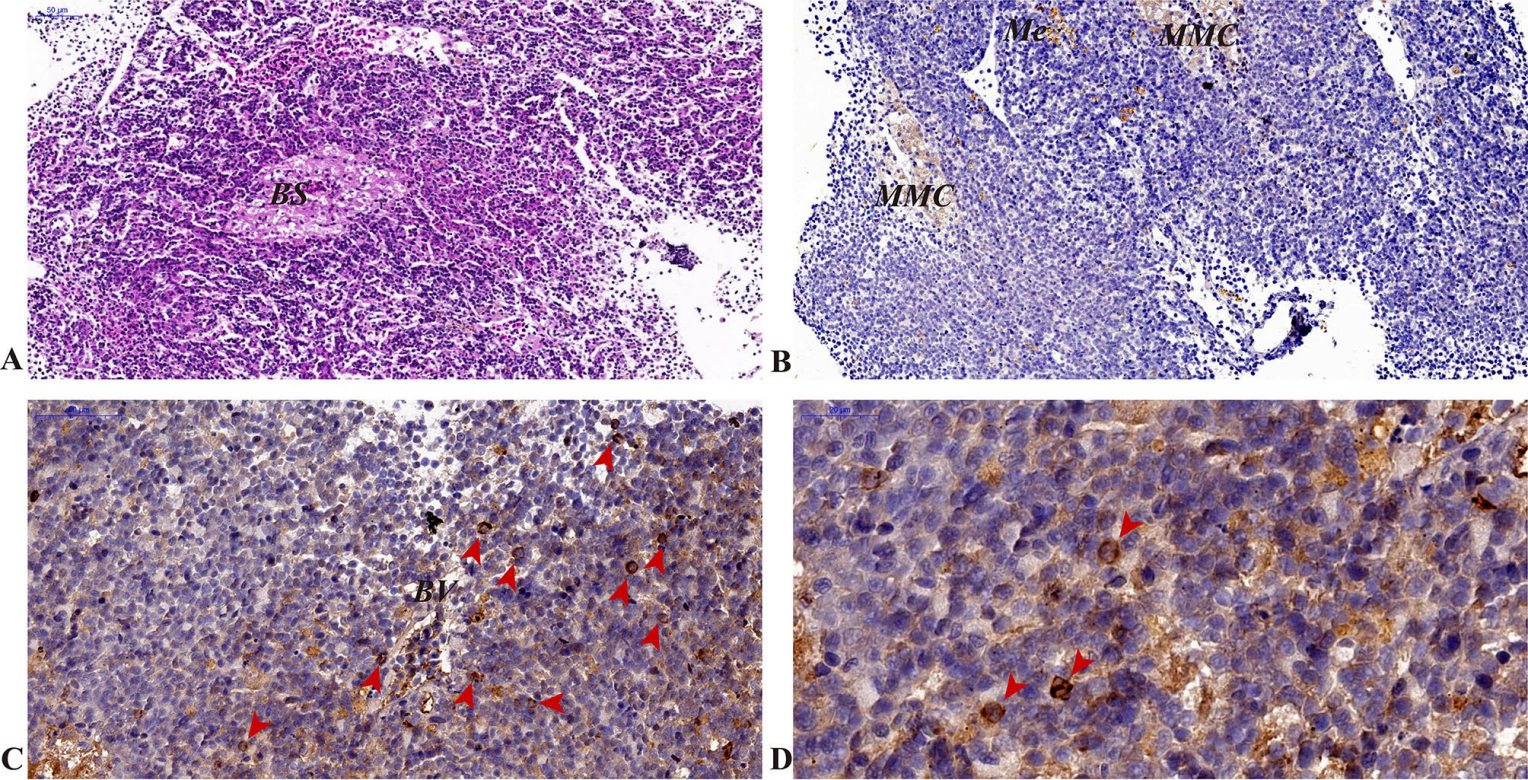
Expression of *Cc*IgZ3 in the head kidney of common carp (**A**) H&E staining, (**B**) isotype control, (**C**) and (**D**) immunohistochemical staining using a polyclonal antibody against common carp *Cc*IgZ3 and counterstaining with haematoxylin. Images showing the MMC (melanocyte macrophage centre), Me (melanocyte), BS (blood sinus), and BV (blood vessel). Scale bars (50 μm and 20 μm) are indicated

**Fig. 3:**
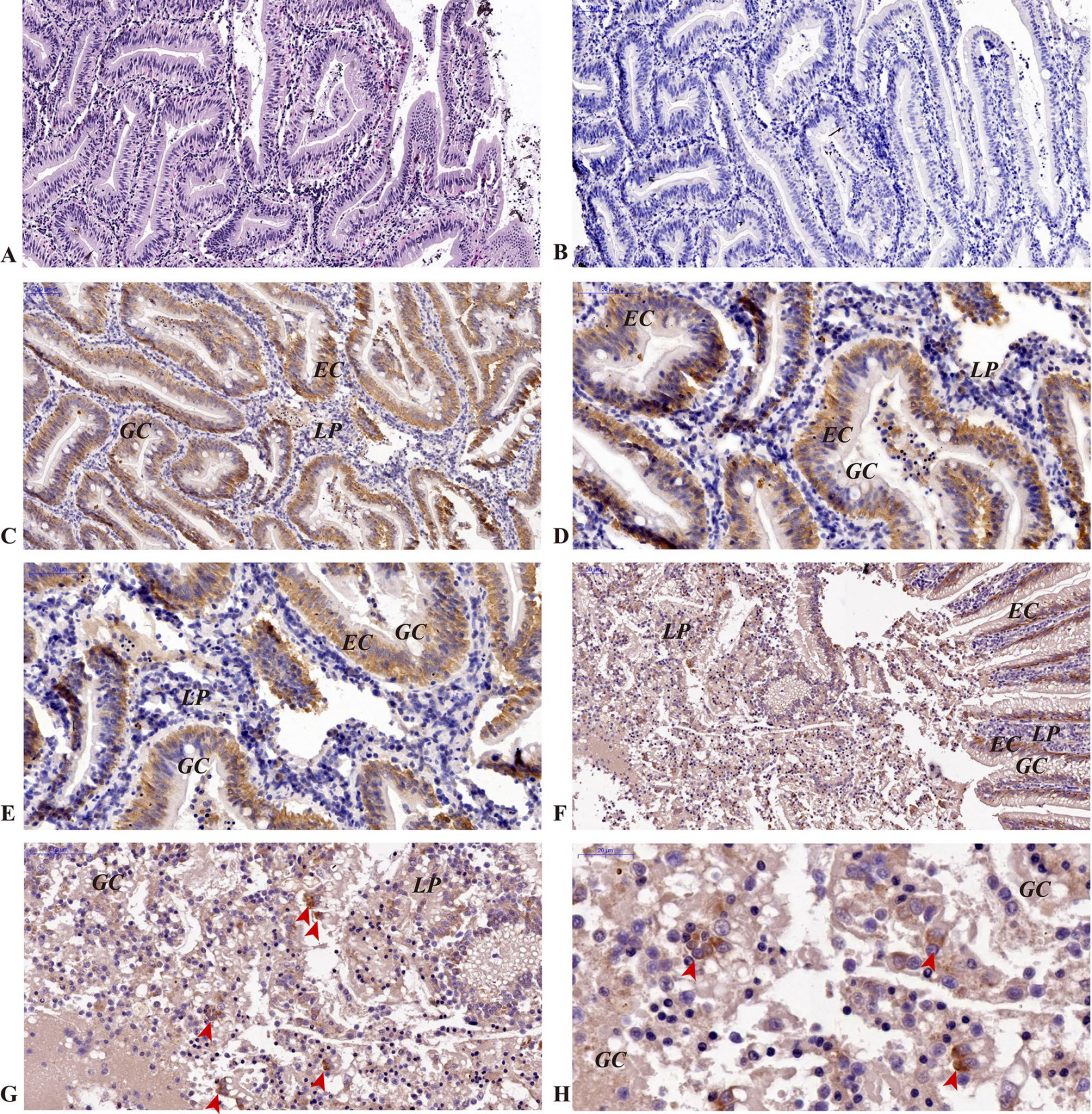
Expression of *Cc*IgZ3 in the hindgut of common carp (**A**) H&E staining, (**B**) isotype control, (**C**) to (**H**) immunohistochemical staining using a polyclonal antibody against common carp *Cc*IgZ3 and counterstaining with haematoxylin. LP, mucosal lamina propria; GC, goblet cell; EC, epithelial cell. Scale bars, 50 μm and 20 μm are indicated

**Fig. 4:**
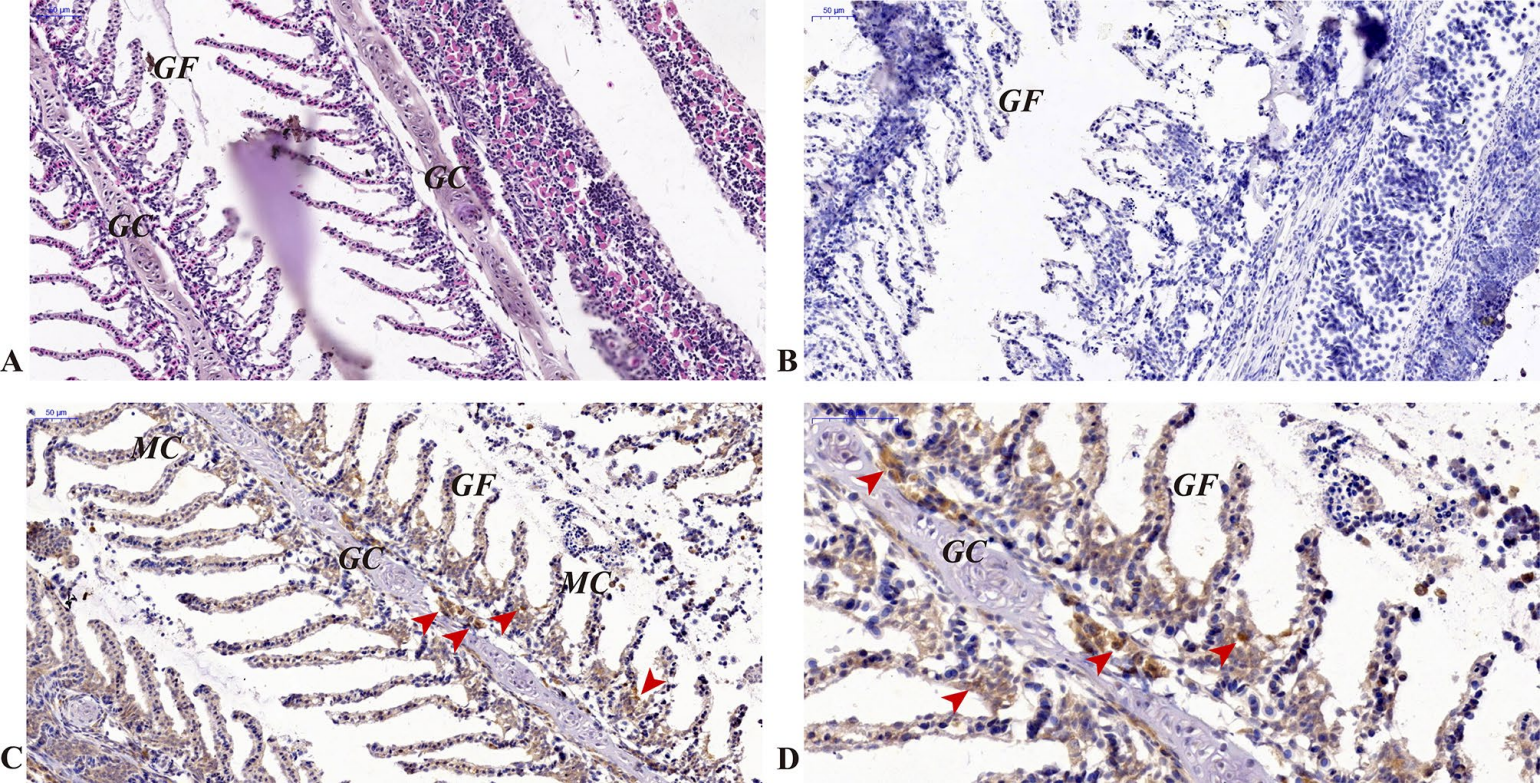
Expression of *Cc*IgZ3 in the gills of common carp (**A**) H&E staining, (**B**) isotype control, (**C**) and (**D**) immunohistochemical staining using a polyclonal antibody against common carp *Cc*IgZ3 and counterstaining with haematoxylin. GF, gill filament; GC, gill chondrocyte; MC, mucosal cell. Scale bars, 50 μm and 20 μm are indicated

Immunohistochemical results revealed expression of the *CcIgZ3* protein in the head kidney, hindgut and gill tissues. In the head kidney, *Cc*IgZ3-positive B cells were distributed and relatively concentrated near blood vessels (Fig. 2, C and 2D). No *Cc*IgZ3-positive B cells were found in melanin macrophage centres (MMC, Fig. 2). The H.E. stain (Fig. 2, A) and isotype control (Fig. 2, B) of the head kidney were performed using serial sections of the head kidney and the results were listed in Fig. 2. In the hindgut of common carp, a wide range of goblet cells were distributed within the intestinal epithelial cells, which can secrete and produce a large amount of mucus. *Cc*IgZ3-positive cells appeared not only among posterior intestinal epithelial cells but also in the mucosa propria, where *Cc*IgZ3-positive B cells were relatively clustered (Fig. 3, C to 3H). The H.E. stain (Fig. 3, A) and isotype control (Fig. 3, B) of the hind gut were performed using serial sections of the hind gut and the results were listed in Fig. 3. In carp gills, there were also many mucous cells between the gill epithelial cells and the CcIgZ3-positive cells on the gill surface. *Cc*IgZ3-positive B cells were found among the gill epithelial cells, and they were relatively concentrated among the gill epithelial cells (Fig. 4, C and 4D). The chondrocytes in the branchial filaments were *Cc*IgZ3 negative (Fig. 4, C and 4D). The H.E. stain (Fig. 4, A) and isotype control (Fig. 4, B) of the gill were performed using serial sections of the gill and the results were listed in Fig. 4.These results indicate that *Cc*IgZ3-positive cells were present in both the head kidney and mucosal immune-associated lymphoid tissues, including the hindgut and gill, and that relatively clustered *CcIgZ3*-positive cells were found in the areas adjacent to the blood vasculature of the head kidney, the mucosal lamina propria of the hindgut and epithelial cells of the gill filament.

### Immunofluorescence co-expression pattern of *Cc*IgZ3 and PCNA in the gills of common carp after TNP-LPS stimulation

**Fig. 5:**
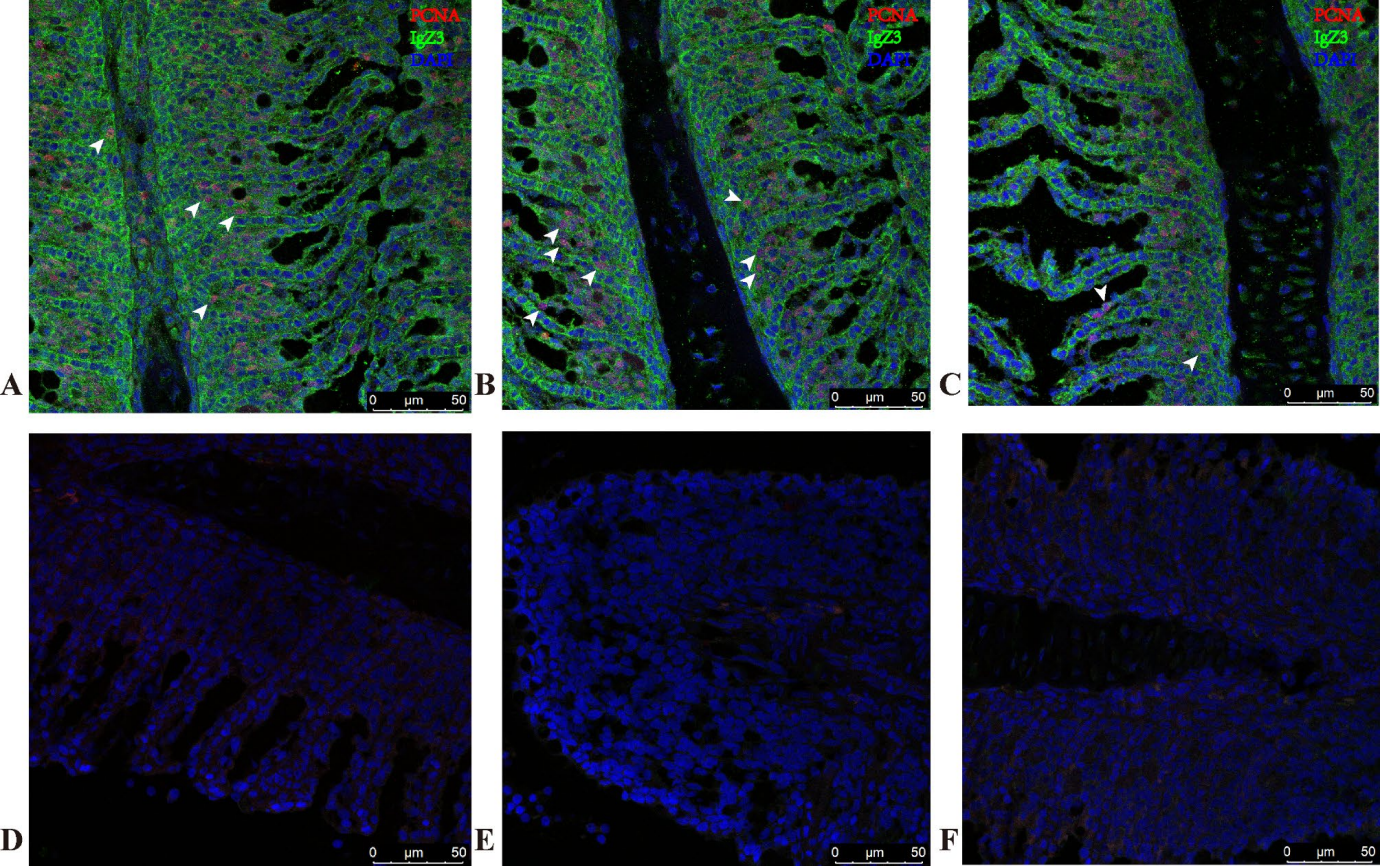
Co-expression of *Cc*IgZ3 with PCNA in the gills of common carp stimulated with TNP-LPS at 3 dps. In common carp, *CcIgZ3* (green) was distributed on the surface of the gill epithelium, and *Cc*IgZ3-positive cells were located between epithelial cells in the gill. PCNA (red) was expressed most in the nucleus (**A** to **C**). The control group was incubated with the isotype antibody (**D** to **F**). The scale bar is indicated

**Fig. 6:**
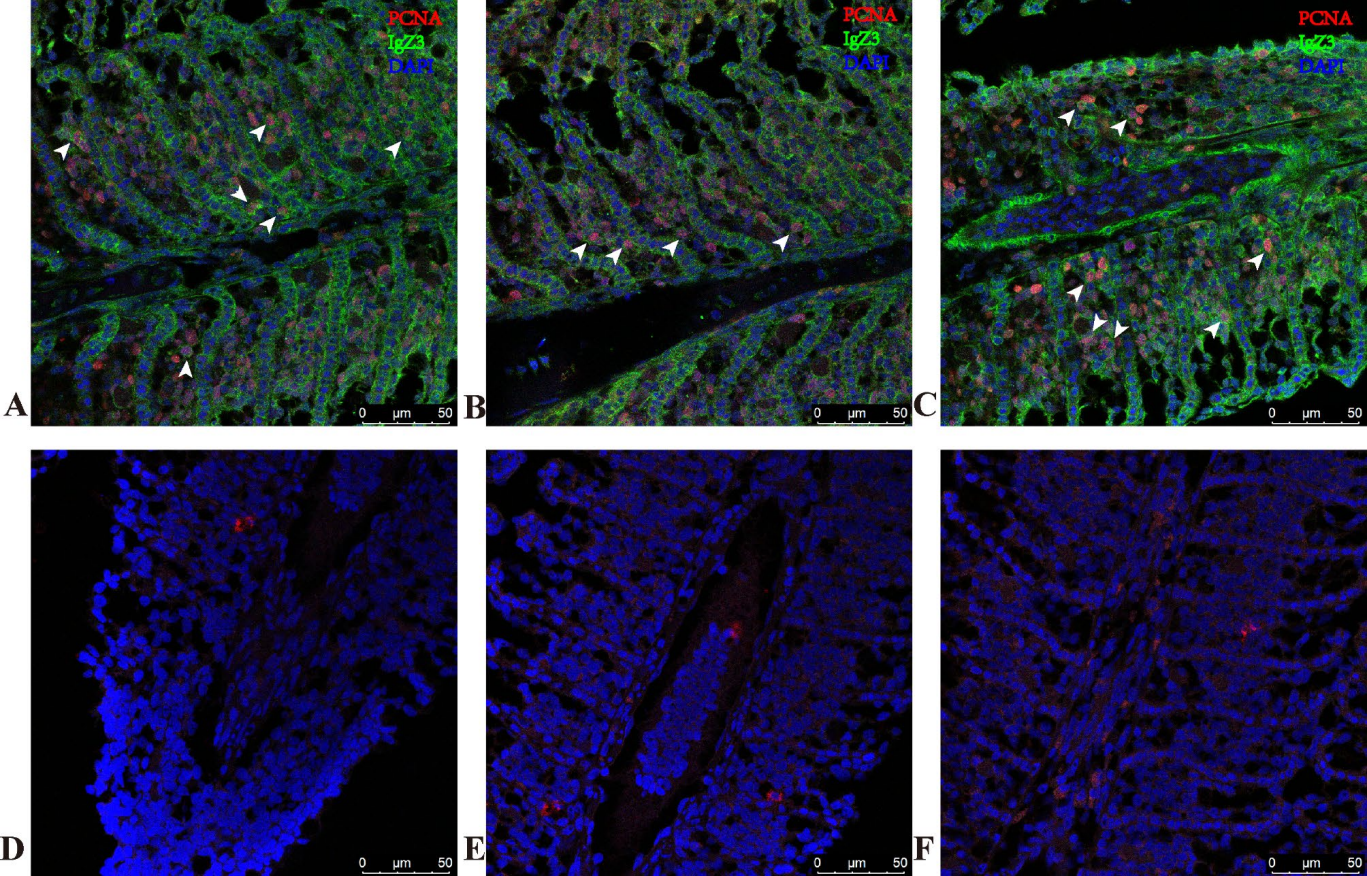
Co-expression pattern of *Cc*IgZ3 with PCNA in the gills of common carp stimulated with TNP-LPS at 7 dps. In common carp, *Cc*IgZ3 (green) was distributed on the surface of the gill epithelium, and *Cc*IgZ3-positive cells were located between epithelial cells in the gill. PCNA (red) was expressed most in the nucleus (**A** to **C**). The control group was incubated with the isotype antibody (**D** to **F**). The scale bar is indicated

To further understand the origin of the *Cc*IgZ3 antibody in mucosal tissues, the distribution and quantity of *Cc*IgZ3 and PCNA double-positive cells in carp gill tissues after TNP-LPS stimulation at 3 dps (Fig. 5, A to 5C) and 7 dps (Fig. 6, A to 6C) were detected by immunofluorescence staining. The results showed that *Cc*IgZ3 and PCNA double-positive cells appeared in the gills of the carp after stimulation, which were distributed among the gill epithelial cells, and that the *Cc*IgZ3^+^PCNA^+^ double-positive cells were much more abundant in the gills of the common carp stimulated with TNP-LPS at 7dps (Fig. 6, A to 6C) than that of 3dps (Fig. 5, A to 5C). The increase of *Cc*IgZ3^+^PCNA^+^ double-positive cells in the gills suggested that TNP-LPS can locally stimulate the proliferation of *Cc*IgZ3^+^ B lymphocytes in gill tissue, which will be helpful for promoting *Cc*IgZ3 antibody secretion and accelerating the response mediated by *Cc*IgZ3 in the gills. In addition, single-positive *Cc*IgZ3 cells and single-positive PCNA cells were also found in the gill tissue. These results indicated that there were non-proliferative *Cc*IgZ3-positive cells and that other cells in the gill may be proliferative. The isotype control was incubated with the isotype antibody (Figs. 5D to 5F and 6D to 6F) .

## Discussion

Bony fish are highly dependent on mucosal immunity. The surface of the fish body is completely covered by the mucosal surface, which includes the gills, skin and guts. The mucosal-associated immune system is widely distributed and plays an important role in fighting various pathogenic infections. Immunoglobulins, including not only IgM but also IgT were widely present in mucosal-associated lymphoid tissues. These immunoglobulins play a role in immune antibacterial, antiviral and antiparasitic responses. IgT is a specific mucosal immune antibody that is expressed in the skin, gill, gut and nasal mucosa and is capable of coating bacteria [4, 10, 24]. However, the knowledge about the production of antibodies in mucosal tissues and the maturation and activation of B cells secreting antibodies are limited.

The mucosal immune system, especially the intestinal mucosal immune system, is thought to have emerged early in the adaptive immune evolution of vertebrates, while structural lymphoid tissue and immunoglobulins were first discovered in the gut of primitive chondrofishes [25]. The mucosal immune system is thought to represent the primitive vertebrate immune system, while the spleen and lymph nodes were later specialized. In humans, the main mucosal immune antibody IgA is produced by plasma cells. In the gut, primordial B-cell precursors of IgA-secreting mucosal plasma cells are activated in Peyer’s patches and mesenteric lymph nodes. B lymphocytes initially activated by antigen can migrate directly to the mucosal lamina propria in this area, but most of these cells leave through lymphatic vessels, pass through the mesenteric lymph nodes, reach the thoracic duct, enter the blood, and selectively re-enter the intestinal mucosa lamina propria via small blood vessels, thereby returning lamina propria B lymphocytes to differentiate into IgA-producing plasma cells. The gut-specific homing of B cells is largely determined by the expression of adhesion molecules on lymphocytes. Unlike in humans, most intestinal IgA-producing B cells in mice develop B-cell activation independent of T cells and class switching, and the IgA antibodies produced in this way have limited diversity, low affinity, and almost no somatic hypermutation.

Approximately 550 million years ago, adaptive immunity emerged in vertebrates. Over the course of evolution, immunoglobulin has evolved into a variety of isotypes [26]. In mammals and birds, IgM, IgG, and IgY isotypes play major roles in systemic immune responses, while IgA is involved mainly in muco-associated immune responses [27]. Early researchers suggested that IgM is the only immunoglobulin type produced in the fish body and in mucosal immunity. However, knowledge of IgT/IgZ, which was first discovered in rainbow trout and zebrafish, has improved our understanding of immunoglobulins in bony fish [4]. With the study of IgT/IgZ in bony fish, an increasing number of researchers have shown that IgT/IgZ mainly plays a role in the mucosal tissues of fish. In addition, researchers have shown that IgT/IgZ is involved in multiple functions of mucosal immunity in different bony fishes. In rainbow trout, IgT is present in the intestinal mucus, where it exists mainly in the form of polymers. IgT can bind to poly-immunoglobulin receptors (pIgRs) and then be transported through intestinal epithelial cells into mucous to perform their functions [4]. In zebrafish, the concentrations of IgT1 were significantly increased in the serum, skin mucus, and gill mucus after bacterial infection, while no expression of IgT2 was detected in the serum, and the concentrations were significantly increased only in the skin mucus and gill mucus [28]. The expression of IgZ in common carp is similar to that in zebrafish. IgZ1 plays a role in systemic immunity, while IgZ2 is mainly expressed in mucosal immune tissues such as the intestine and gill [18]. In previous studies, we identified a new subtype of common carp IgZ, *Cc*IgZ3, which is widely expressed in the head kidney, gill, spleen and gonads. *Cc*IgZ3 expression can be induced by *Aeromonas hydrophila* infection via intraperitoneal injection and soaking [29]. In this study, we further investigated the immune response of *Cc*IgZ3 following induction by TI and TD antigens, and we further investigated proliferating *Cc*IgZ3^+^ B lymphocytes in the gills.

LPS is one of the main components of gram-negative bacteria. As one of the most widely studied TI antigens in mammals, LPS is recognized by B cells through B-cell receptors and Toll-like receptors [30]. The two typical receptors of LPS, TLR4 and CD14, are not expressed on human B cells, so LPS is not a mitogen of human B cells [31, 32]. In contrast, by expressing TLR4 on mouse B cells, LPS can stimulate B cells in the mouse abdominal cavity and spleen to enter the S phase and G2/M phase [30]. The immune system of bony fish is very different from that of mammals. Instead of bone marrow or the typical lymph nodes, bony fish use the anterior kidney as the primary haematopoietic site, and in the absence of immune activation, the anterior kidney is the primary storage site for Ig-secreting cells [33]. In rainbow trout, populations of mature B cells, proliferating B cells, and plasma cells are present in the head kidney, blood, and spleen; these cells have high plasma cell production capacity after in vitro LPS induction of resting B cells, while the blood has almost no such cells [34]. Moreover, LPS can significantly induce the proliferation of IgM^+^ B cells in trout [35]. In grass carp, LPS can induce the proliferation and secretion of IgM^+^ B cells by significantly upregulating the expression of IL-21 [36]. These results are also consistent with our findings. After immunofluorescence double labelling of *Cc*IgZ3 and PCNA, double-positive cells and their respective single-positive cells were observed; the labelling experiments indicated that some cells among the *Cc*IgZ3-positive cells exhibited proliferative activity under LPS stimulation, indicating their activation in mucosal tissues. In conclusion, LPS stimulation can promote the proliferation and differentiation of fish B lymphocytes.

Early studies of mucosal immunity in fish have shown that immunoglobulins present in the skin and bile are not the product of serum transport or active transport but are the result of local mucosal synthesis, and vaccination studies have shown that the mucosal system of fish can be stimulated to produce antibodies and that these antibodies can be produced in the absence of a systemic immune response [37]. Lymphocytes have been found in different mucosal sites in a variety of animals. How do B cells in the mucosal immune tissue of bony fish proliferate and secrete antibodies after antigen activation? The antigen-activated B cells can be better determined based on the expression of proliferation-associated nuclear antigen (PCNA) in mucosal B cells. PCNA is a proliferating-associated nuclear antigen that is an important factor involved in DNA replication and repair. PCNA slides around DNA in the form of a homotrimer ring, anchoring DNA polymerase and other DNA editing enzymes. Moreover, PCNA also interacts with regulatory proteins, such as p15, through the sequence motif of the PIP-box (PCNA interaction protein box), and is involved in the regulation of DNA replication, DNA damage repair, and cell cycle progression [38]. Changes in PCNA posttranslational modifications and the inhibition of PCNA interaction with regulatory proteins affect cell proliferation [39]. As a nonhistone nucleoprotein, PCNA was highly conserved between species during evolution and is widely used as a marker of cell proliferation in various tissues in fish. These include *Oreochromis niloticus* gills [40], *Psetta maxima* brain [41], olfactorysyste [42], goldfish retina [43], and *Sparus auratus* bone tissue [44].

The expression of PCNA is related to cell proliferation, and PCNA can also be observed in the growing follicles of fish [45]. The oocytes in these studies were in meiotic arrest and did not replicate nuclear DNA. The expression of PCNA in the nucleus is related to the function of PCNA as a cofactor of DNA polymerase that participates in DNA repair and modification and is an important factor in ensuring normal follicle development. In mammals, studies have shown that PCNA is involved in class switching during the maturation and differentiation of B cells, antibody affinity maturation and effector cell generation [39]. Ubiquitination of lysine residues at site 164 in PCNA plays an important role in antibody class conversion and antibody diversity [39]. In chicken B-cell DT40, mono-ubiquitination of lysine residues at this site mediates the recruitment and activation of polymerase in the immunoglobulin variable region to promote the generation of DNA mutations [39]. In mice, after the lysine at this site was mutated to arginine, the number of somatic cells with mutated AT bases in the IgV region was significantly reduced [39]. However, studies on the function of PCNA in the maturation and differentiation of fish B cells are rare. Fish were the first vertebrates to produce lymphocytes and specific immunity. Understanding the differentiation process of lymphocytes is highly important for understanding the evolution of adaptive immunity.

## Conclusion

The results in the current research showed that TNP-LPS could activate the *Cc*IgZ3 response and *Cc*IgZ3^+^ B lymphocytes could proliferate in gills of the common carp. These results suggest that an effective vaccine can be designed to promote production of the mucosal antibody *Cc*IgZ3.

## Data Availability

The datasets used during the current study are available from the corresponding author upon reasonable request.
